# The Biocompatibility of Dental Graded Nano-Glass-Zirconia Material After Aging

**DOI:** 10.1186/s11671-018-2479-4

**Published:** 2018-02-23

**Authors:** Ting Sun, Ruoyu Liu, Xiangning Liu, Xiaoli Feng, Yanli Zhang, Renfa Lai

**Affiliations:** 1 Medical Center of Stomatology, The First Affiliated Hospital of Jinan University, Guangzhou, 510630 China; 2 Shenzhen Traditional Chinese Medicine Hospital, Shenzhen, 518033 China; 3 Department of Stomatology, Nanfang Hospital, Southern Medical University, Guangzhou, 510515 China

**Keywords:** Dental, Zirconia, Graded, Aging, Biocompatibility

## Abstract

**ᅟ:**

A graded nano-glass/zirconia (G/Z) system has been developed via the infiltration of nano-glass into a nano-zirconia surface, which is advantageous for robust core-veneer bonds. The aging issue is a key for yttrium-stabilized tetragonal zirconia polycrystals (Y-TZPs), and therefore, it is necessary to evaluate the influence of aging degradation on the biocompatibility of G/Z systems before their possible clinical application. Herein, such biocompatibility testing was performed with human gingival fibroblasts (HGFs) seeded onto unaged/aged G/Z and Y-TZP for 2–72 h. Assessments included an oral mucous membrane irritation test in conjunction with analyses of cell viability, cell adhesion, and oxidative stress responses. Significant metabolic decreases in aged G/Z- and Y-TZP-treated cells were observed at 72 h. G/Z did not elicit any significant differences in cell viability compared with Y-TZP over 72 h both before and after aging. The oxidative stress data for the aged G/Z- and Y-TZP-treated cells showed a significant increase at 72 h. The G/Z specimens did not elicit any significant differences in ROS production compared with Y-TZP over 72 h both before and after aging. The cell adhesion rates of both G/Z and Y-TZP increased significantly after aging. The cell adhesion rates of G/Z and Y-TZP were not significantly different before and after aging. According to the oral mucous membrane irritation test, scores for macroscopic and microscopic observations for both the aged G/Z and unaged G/Z sides were 0, demonstrating no consequent irritation.

**Conclusions:**

The excellent biocompatibility of G/Z indicates that it has potential for future clinical applications.

## Background

Dental zirconia-based ceramics (e.g., 3 mol% yttrium-stabilized tetragonal zirconia polycrystals (3Y-TZPs)) exhibit excellent mechanical strength and superior fracture resistance due to inherent transformation toughening mechanisms, and they are widely utilized for the fabrication of prosthetic devices [1]. Zirconia core materials are usually coated with translucent veneering porcelain to cover their opaque appearance. However, layered zirconia restorations tend to fail; chipping and delamination of the veneering ceramic have been reported as the most frequent reason for the failure of zirconia-based restorations [2, 3]. Chipping and delamination of the veneering ceramic were reported to result from mismatches of the thermal expansion coefficient and elastic modulus between the zirconia cores and veneering ceramics [4]. Consequently, in our previous study, we introduced a new concept for the improvement of core-veneer bonding by infiltrating a low modulus nano-sized glass with a matching thermal expansion coefficient into the zirconia surface sintered from nano-zirconia particles, thus producing elastic graded nano-glass/zirconia (G/Z) systems. The bond strengths of the G/Z systems to veneering porcelains were demonstrated to be threefold higher than those of conventional zirconia-based systems [4].

The aging of Y-TZP is well acknowledged. The aging of Y-TZP can be induced by an oral environment, with exposure to humidity, mechanical loading, and low temperature, resulting in surface roughening, microcracks, and Y-TZP particle release into the body [5, 6]. In the presence of humidity and low temperature, a tetragonal to monoclinic (t-m) zirconia phase transformation could be triggered. The crystal volumetric expansion results in localized stress and microcracking in the material surface, allowing water to further penetrate in the interior of the material, leading to additional phase transformation and resulting in the degradation of the mechanical properties [7–9]. Additionally, it is now widely known that the physicochemical properties of a biomaterial such as surface roughness and chemical composition have an influence on its biocompatibility. Thus, it is necessary to evaluate the influence of aging degradation on the biocompatibility of G/Z.

Zhang et al. [10, 11] infiltrated glass into a dense zirconia substructure and developed a graded glass-zirconia composite with superior mechanical properties. However, the biocompatibility of the graded glass-zirconia composite is unknown, especially with the consideration of aging phenomenon.

Consequently, biocompatibility testing of the newly developed G/Z system is essential for its clinical application due to the addition of glass materials and the consequent structural changes. The induction of the G/Z system may provide a solution for failures of zirconia-based restorations and thus improve their success rates. Therefore, the biocompatibility testing of the G/Z system before and after aging will provide guidelines on biosafety for the clinical application of G/Z.

In the present study, the biocompatibility of the G/Z system before and after aging was evaluated. Assessments involved an oral mucous membrane irritation test in conjunction with analyses of the cell viability, cell morphology, cell adhesion, and oxidative stress responses.

## Methods

### Preparation of Specimens

Y-TZP is a biocompatible material already approved for clinical applications, and herein, Y-TZP specimens were set up as the control group. All specimens were produced as uniform plates (1.5 × 1.5 × 0.2 cm). ISO 13356 describes the evaluation of tested specimens with a simplified geometry (bending bars) and a polished surface.

#### Preparation of G/Z Specimens

Glass powders were milled until nanosized particles were obtained with a nanometer grinding instrument (Emax, Retsch, Haan, North Rhine-Westphalia, Germany). The main components and percentages (> 1 wt%) of the infiltrating glass are listed in Table 1 [4]. Yttrium-stabilized zirconia powders (5.18 wt% Y_2_O_3_, TZ-3Y-E grade; Tosoh, Tokyo, Tokyo Prefecture, Japan) were compressed under a uniaxial pressure of 150 MPa for 2 min and were then partially sintered at 1350 °C for 2 h in a muffle furnace. The desired oxides were ball-milled into 200-mesh powders. The Y-TZP substrate specimens were presintered at 1200 °C for 2 h, forming porous structures. The melted glass slurries were applied onto the top surface of the presintered Y-TZP porous substrate specimens. The coated specimens were then infiltrated at 1350 °C for 2 h to produce a graded glass-zirconia structure. Glass infiltration and densification were carried out simultaneously.

**Table 1:** Chemical composition of graded nano-glass-zirconia material

Material	Manufacturer	Main components (wt%)
Glass composition		La_2_O_3_ 20.0; SiO_2_ 20.0; B_2_O_3_ 15.0; BaO 15.0; Al_2_O_3_ 10.0; ZrO_2_ 5.0; Y_2_O_3_ 5.0; TiO_2_ 4.0; CaO 4.0 [5]
Y-TZP powder	Tosoh, Tokyo, Japan	Y_2_O_3_ 5.18, ZrO_2_ 94.82 [5]

#### Preparation of Y-TZP Specimens

Y-TZP blanks (Weiland, Weiland Dental, Pforzheim, Baden-Württemberg, Germany) were designed, milled, and sintered to full density using a CAD/CAM system (Zenostar, Weiland Dental, Pforzheim, Baden-Württemberg, Germany).

### Cell Culture

Human gingival fibroblasts (HGFs) were cultured in Dulbecco’s modified Eagle’s medium (DMEM, Nutrient Mixture F-12) containing 10% fetal bovine serum, 1% penicillin/streptomycin, 1% l-glutamine, and 1% non-essential amino acids in a humidified atmosphere of 5% CO_2_ at 37 °C. The medium was changed every 3 days. Cells were removed from culture dishes by rinsing in phosphate-buffered saline (PBS) and incubated in a trypsin-EDTA solution. Cells were seeded on each test substrate at 1 × 10^5^ cells/mL in the same media for all assays.

### Aging

To stimulate the chewing conditions, mechanical aging was performed in artificial saliva at 37 °C, and the load was applied using a three-point flexure fixture at a 2 Hz frequency. The following aging profiles were used: 80 N load and 10^5^ cycles for all specimens [12, 13].

### Cell Viability

The viability of HGF following unaged and aged G/Z and Y-TZP exposure was determined at 2, 24, 48, and 72 h (exposure time) using the alamarBlue^®^ salt assay as a 10% solution in DMEM. Before the assay test, all specimens were removed from the HGF, and then, 500 μL of alamarBlue^®^ dye was added, followed by incubation for 4 h. Aliquots (100 μL) were decanted into 96-well cell culture dishes, and fluorescence intensity was determined at excitation (530 nm) and emission (580 nm) wavelengths with a Synergy™ H4 Microplate Spectrophotometer (BioTek, Winooski, Vermont, USA). All experiments were performed in triplicate on three occasions. The cell viability was calculated as follows: viability (%) = (absorbance of the treated wells) / (absorbance of the control wells).

### Oxidative Stress

Reactive oxygen species (ROS) levels of G/Z- and Y-TZP-treated HGF before and after aging were identified with chemiluminescence using the Reactive Oxygen Species Assay Kit (Nanjing Jiancheng Bioengineering Institute, Nanjing, Jiangsu).

### Cell Adhesion

HGFs were cultured for 2 h on G/Z and Y-TZP specimen surfaces before and after aging. After fixation, cell nuclei were stained with 4′,6-diamidino-2-phenylindole dihydrochloride (DAPI) (Yeasen, Shanghai, Shanghai District, China). Images were obtained with an inverted LSM 510 fluorescence microscope (Carl Zeiss, Jena, Tuttlingen, Germany). The adhered cells were analyzed in randomly selected areas in five sections (450 μm × 450 μm) at a magnification of × 200. The cell adhesion rates were determined through the number of adhered cells divided by the total number of seeded cells.

### Cell Morphology

HGFs were cultured for 2 h on unaged and aged G/Z specimen surfaces before and after aging. After fixation, the cells were stained for filamentous actin (F-actin) using rhodamine phalloidin (1:100 in 3% BSA in PBS). Images were obtained with an inverted LSM 510 fluorescence microscope (Carl Zeiss, Jena, Tuttlingen, Germany). Samples were mounted on glass coverslips using DAPI (Yeasen, Shanghai, Shanghai District, China) for the visualization of cell nuclei. The cell morphologies on G/Z and Y-TZP surfaces before and after aging were also observed via scanning electron microscopy (SEM) with a XL-30 ESEM (Philips, Eindhoven, North Brabant, The Netherlands).

### Oral Mucous Membrane Irritation Test

The oral mucous membrane irritation test was conducted according to YY/T 0127.13-2009 medicine standards of the People’s Republic of China. Ten Wistar male mice were selected for this test. The aged G/Z specimen was placed in one cheek pouch for each animal as the tested material, while the unaged G/Z specimen was placed in the contralateral side as the control. The animals were sacrificed after 2 weeks, and the pouches were examined macroscopically following removal of the disks. Histological analyses of the buccal mucosa were further performed on cryosections that were stained with hematoxylin and eosin. The average grades for all macroscopic and microscopic observations were obtained. The control group average was subtracted from the test group average to yield the irritation index.

### Statistical Analyses

One-way analysis of variance (ANOVA) was used for the pooled (all exposure times) cell viability, oxidative stress, and cell adhesion rate data for the assessment of individual dental specimens (SPSS 22.0; SPSS Inc., Chicago, IL, USA).

## Results

### Graded Layer Structure

**Fig. 1:**
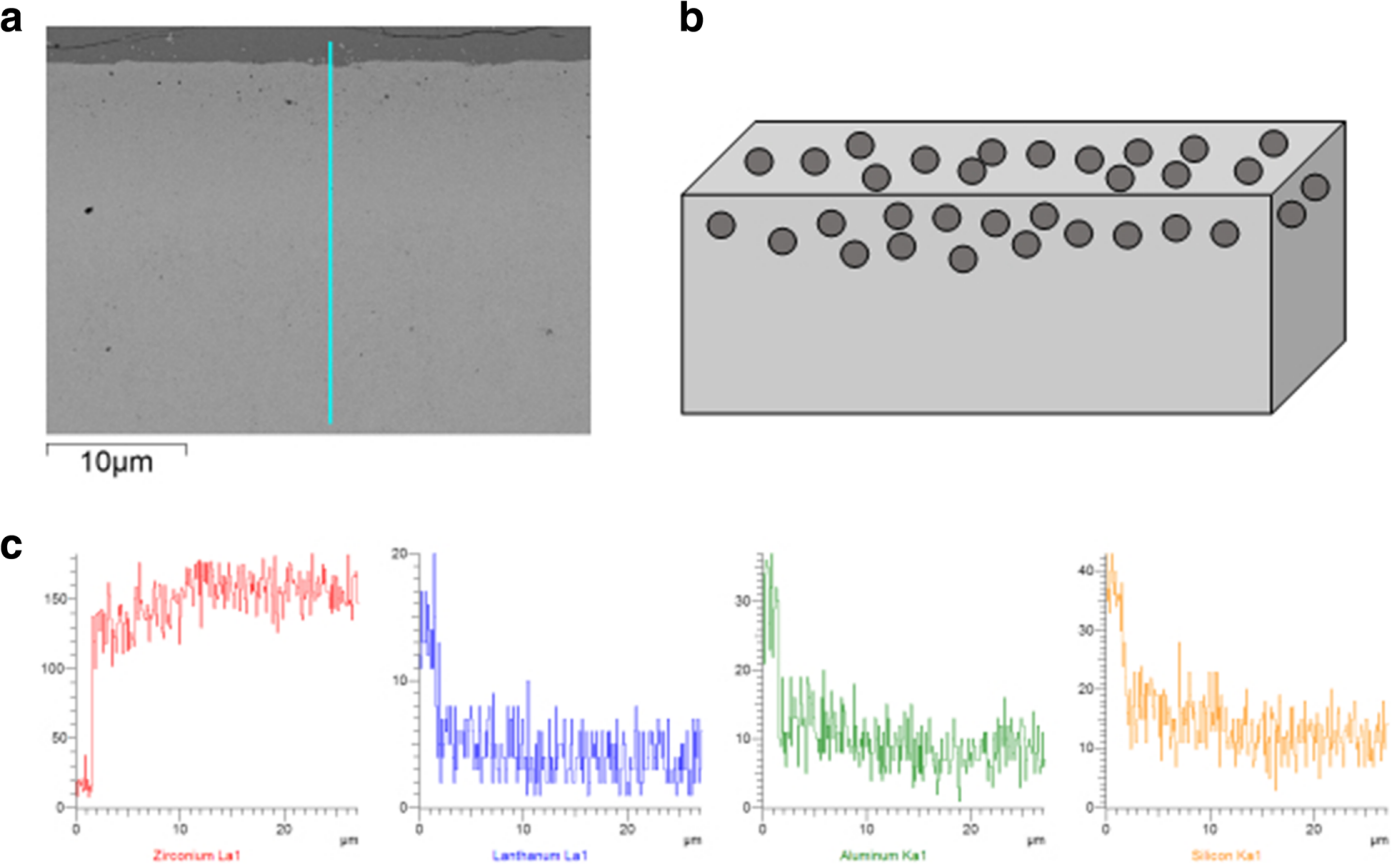
Physical and chemical properties of G/Z. **a** Structural diagram. **b** SEM image. **c** EDS analysis of the functionally graded layer

The thickness of the graded layer was controlled to be approximately 0.9–1.0 mm. The structure and SEM images of the G/Z system are shown in Fig. 1a, b. Figure 1a, b depicts a morphology consisting of traces of residual glass, glass-coated zirconia grains, and intergranular voids, which created a surface morphology ideal for increasing the core-veneer bond strength. Furthermore, EDS analysis of the graded layers is shown in Fig. 1c, showing with the increase in the distance from the surface, the content of the Zr element increased while the contents of the Si, Al, and La elements decreased. Details have been described in our previous study [4].

### Cell Viability

**Fig. 2:**
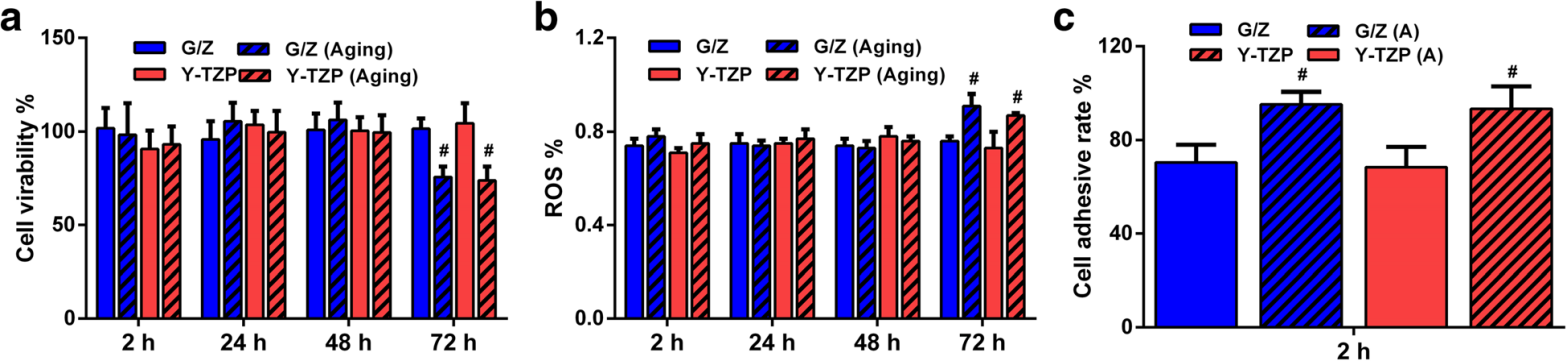
Biocompatibility of G/Z and Y-TZP before and after aging. Data represent the mean ± SD, *n* = 5. **a** Cell viability of aged and unaged specimen-treated HGF. **b** Ros production of aged and unaged specimen-treated HGF. **c** Cell adhesive rates of aged and unaged specimen-treated HGF. Significance versus control group: ^#^*P* < 0.01; ^*^P < 0.05

Significant metabolic decreases in aged G/Z- and Y-TZP-treated cells were observed at 72 h (*P* < 0.00001) (Fig. 2a). No significant metabolic decrease in aged G/Z-treated cells was observed at 2 h (*P* = 0.47), 24 h (*P* = 0.82), and 48 h (*P* = 0.53) (Fig. 2a). No significant metabolic decrease in aged Y-TZP-treated cells was observed at 2 h (*P* = 0.82), 24 h (*P* = 0.32), and 48 h (*P* = 0.54) (Fig. 2a). The G/Z specimens did not elicit any significant differences in cell viability compared with Y-TZP at 2 h (*P* = 0.94), 24 h (*P* = 0.86), 48 h (*P* = 0.68), and 72 h (*P* = 0.61) of exposure before aging. The G/Z specimens did not elicit any significant differences in cell viability compared with Y-TZP at 2 h (*P* = 0.98), 24 h (*P* = 0.54), 48 h (*P* = 0.73), and 72 h (*P* = 0.50) of exposure after aging.

### Oxidative Stress

Oxidative stress data for the aged G/Z- and Y-TZP-treated cells showed a significant increase at 72 h (*P* < 0.00001, Fig. 1b). In contrast, aged G/Z-treated cells elicited no significant difference in ROS production at 2 h (*P* = 0.91), 24 h (*P* = 0.42), and 48 h (*P* = 0.62). Additionally, aged Y-TZP- treated cells elicited no significant difference in ROS production at 2 h (*P* = 0.07), 24 h (*P* = 0.40), and 48 h (*P* = 0.53). The G/Z specimens did not elicit any significant difference in ROS production compared with Y-TZP at 2 h (*P* = 0.16), 24 h (*P* = 0.79), 48 h (*P* = 0.14), and 72 h (*P* = 0.43) of exposure before aging. The G/Z specimens did not elicit any significant difference in ROS production compared with Y-TZP at 2 h (*P* = 0.27), 24 h (*P* = 0.17), 48 h (*P* = 0.07), and 72 h (*P* = 0.15) of exposure after aging.

### Cell Adhesion

**Fig. 3:**
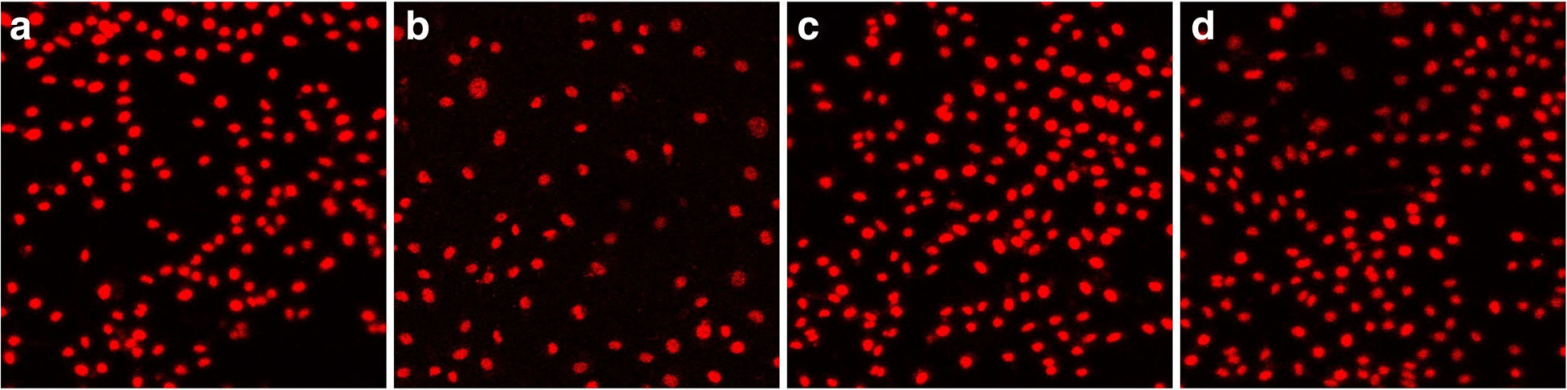
Cell adhesion to G/Z and Y-TZP before and after aging. **a** Aged G/Z. **b** Unaged G/Z. **c** aged Y-TZP. **d** Unaged Y-TZP

The cell adhesion rates of both G/Z and Y-TZP increased significantly after aging (Fig. 2c). The cell adhesion rates of unaged G/Z and Y-TZP were not significantly different (*P* = 0.71) (Fig. 2c). The cell adhesion rates of aged G/Z and Y-TZP were not significantly different (*P* = 0.71) (Fig. 2c). The cell adhesion rates of G/Z and Y-TZP showed no significant differences after aging (*P* < 0.00001) (Fig. 2c). Characteristic photographs of cell adhesion on Y-TZP and G/Z before and after aging are shown in Fig. 3a–d.

### Cell Morphology

**Fig. 4:**
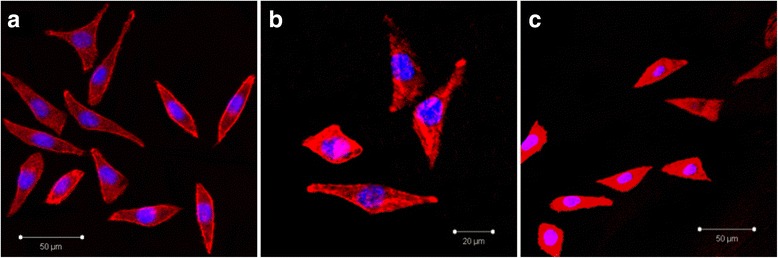
Attachment, spreading, and morphology of HGF on G/Z before and after aging observed with fluorescence microscopy. **a**, **b** Aged G/Z. **c** Unaged G/Z. Cells were cultured for 72 h on substrates and then fixed and stained for filamentous actin (F-actin, red) and nuclei (blue)

Fluorescence images at different incubation times showed that cells were attached to G/Z surfaces; however, spreading was greater on aged G/Z surfaces (Fig. 4a–c), where the cells were flattened and well spread with a polygonal shape.

**Fig. 5:**
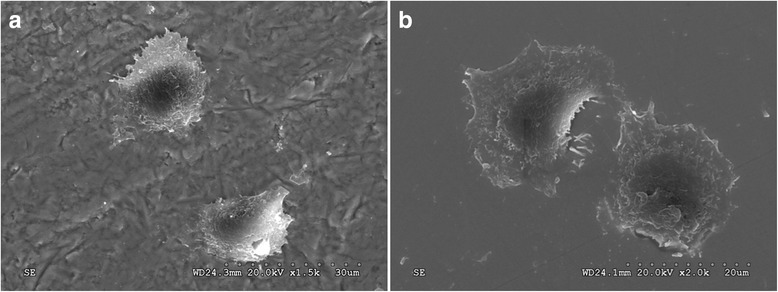
SEM micrographs of the HGF morphology on G/Z before and after aging 72 h post culture. **a** Aged G/Z. **b** Unaged G/Z. Original magnification: × 2000

SEM images showed that cells cultured on aged and unaged G/Z surfaces were considerably flattened with extensions or elongated bodies and numerous microvilli (Fig. 5a, b). Rounded nuclei can be observed, confirming the attachment of the spread of cell cytoplasm to the specimen surface (Fig. 5a).

### Oral Mucous Membrane Irritation Test

**Fig. 6:**
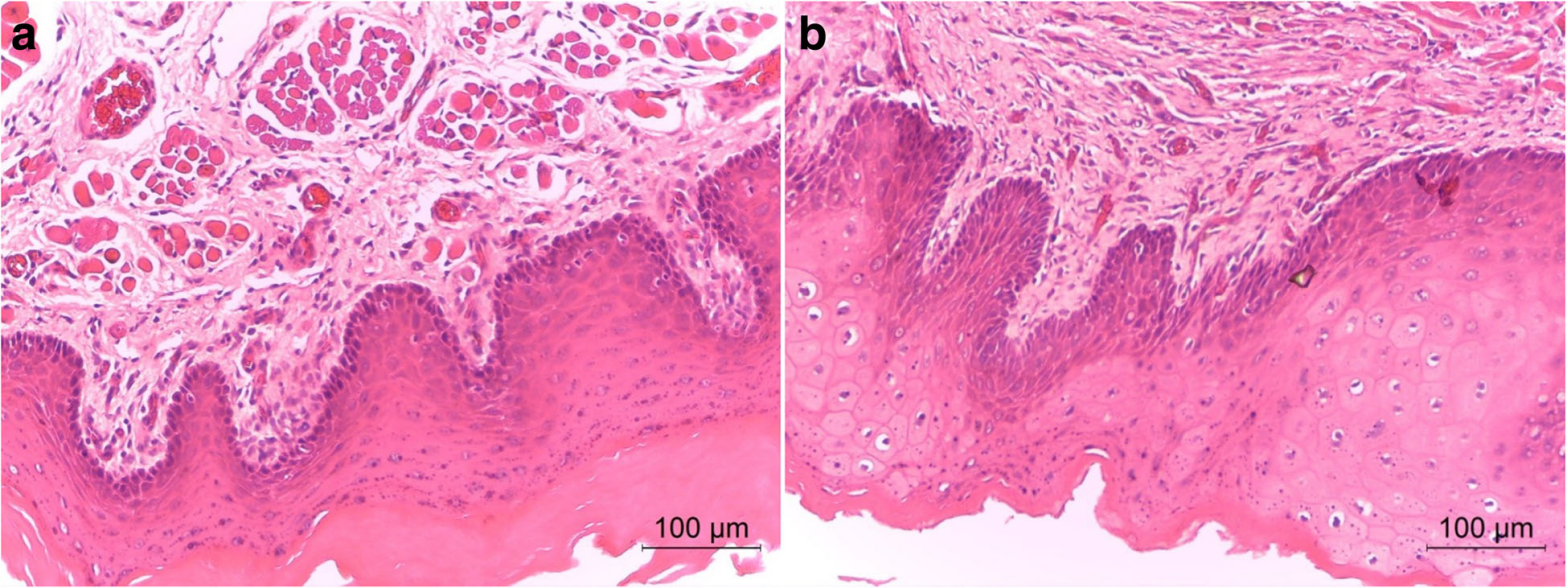
Pathological examination of the mucosa treated with aged G/Z (**a**) and unaged G/Z (**b**)

Scores for the macroscopic observations for both the testing and contralateral sides were 0, demonstrating no consequent irritation. Additionally, scores from the microscopic evaluation for both sides were 0, indicating no apparent irritation reaction. Figure 6a, b demonstrates that no histopathological changes were observed in the buccal mucosa treated with unaged G/Z and aged G/Z.

## Discussion

Metal ceramic materials have been increasingly replaced by metal-free materials as the release of metal ions has been widely discussed. Various metal ions including silver [14], gold [15], titanium [16], and nickel [17] of dental prosthesis could be released into the saliva and plasma. McGinley et al. even reported that diffused Ni ions from a dental Ni-Cr alloy could spread throughout the epithelial tissue to the basal lamina and subsequently throughout the extracellular matrix, resulting in a loss of cell viability and tissue integrity [18]. Present studies mostly focused on the development and improvement of all ceramic materials. Therefore, G/Z was introduced in our previous study [4] for the improvement of the success rates of zirconia-based materials. However, the biocompatibility of the G/Z system with the consideration of aging was unknown. Biocompatibility tests and moderate controls are essential. Consequently, a series of biocompatibility tests was conducted and compared with the *gold standard*, Y-TZP, with the consideration of aging. Furthermore, the surface topography as well as physical and chemical properties have been proven to be influential for cell adhesion and viability by studies [19]. All specimens were therefore sandblasted and polished to a clinical surface roughness.

Significant metabolic decreases in aged G/Z- and Y-TZP-treated cells were observed at 72 h (Fig. 2a), proving that aging decreases the cell proliferations for G/Z and Y-TZP. The influence of aging on the biocompatibility of zirconia materials is controversial. A previous study reported the decreased biocompatibility of zirconia after aging [20]. Meanwhile, a recent study proved the increase in the biocompatibility of aged zirconia [21]. The different influence of aging on the biocompatibility might result from different aging procedures, including the cycle, temperature, load, and frequency [22]. The influence of aging on zirconia’s physical and chemical property changes depends on the aggressiveness of the aging procedure for the degradation of zirconia. To simulate long-term intra-oral conditions, the aging procedure used in this study was based on clinical parameters, such as the bite load and frequency, the use of a humid environment, and the temperature of the human body [22].

Cell viability relies on mitochondrial activity. The decrease of cell proliferation and the increase of ROS production might be attributed to the diffused ions spreading throughout the epithelial tissue to the basal lamina and subsequently throughout the extracellular matrix, resulting in a loss of cell viability and tissue integrity [6, 23].

The cell adhesion rates of both G/Z and Y-TZP increased after aging (Fig. 2c). Characteristic photographs of cell adhesion on Y-TZP and G/Z before and after aging are shown in Fig. 3a–d. Precise observation of cell attachment on G/Z was conducted. Double-labeled fluorescent staining (Fig. 4a, b) and SEM views (Fig. 5a, b) demonstrated that cells cultured on both aged and unaged G/Z were flattened and well spread.

Cell adhesion depends on the physicochemical properties of a biomaterial. It is well acknowledged that migration and adhesion are biological parameters that are not necessarily directly linked. Cells can migrate slowly with very high adhesion [24, 25]. Al Qahtani et al. [26] also reported that the sandblasted surface of Y-TZP presented higher cell adhesion but low cell proliferation when incubated with Saos-2 osteoblasts. The surface wettability is a factor that also determines the preference of cell adhesion, through regulation of amounts of the protein adsorbed on the surface [27]. It was reported that the cells on a superhydrophilic surface even started proliferation as soon as adhesion was complete, and this phenomenon was highly related to the high amounts of the protein adsorbed on the hydrophilic surface [28]. The aging abrasion of G/Z and Y-TZP provides rough surfaces with strong wettability, allowing the strong adhesion of cells. This type of surface will be optimal for gingival adhesion around for dental abutment surfaces. In contrast, smooth surfaces give restricted adhesion properties to the materials, as appropriate for surfaces designed to prevent biofilm formation in the septic environment of the mouth [29]. As dental prosthesis materials, aging abrasion of G/Z and Y-TZP therefore increased the probability for biofilm formation. The cell adhesion rates of G/Z and Y-TZP showed no significant differences before and after aging (Fig. 2c). This finding proved that G/Z and Y-TZP exhibit similar cell attachment properties before and after aging, indicating the promising surface biological properties of G/Z.

In vivo irritation tests are critical for the long-term application of oral medical devices. Herein, no macroscopic or microscopic pathological changes were observed for G/Z-treated mucosa (Fig. 6a, b).

The existence of a large amount of *m*-ZrO_2_ could result in a decrease in the strength of zirconia. The reliable biocompatibility of the G/Z system might be attributed to the small phase change during the infiltration procedure, which was proven in our previous study [4]. Another study proved the fair aging resistance of infiltrated Y-TZP materials. Inokoshi et al. [30] reported that Al_2_O_3_-infiltrated Y-TZP was hydrothermally stable after aging thanks to a high amount of c-ZrO_2_ phase at the interlayer surface, although it has a higher initial monoclinic volume fraction compared to the Y-TZP.

Several studies confirmed the reliable biocompatibility of the glass-zirconia composition. L-929 fibroblast- and Saos-2 osteoblast-like cells presented good adhesion and proliferation on the surface of HAp-Al_2_O_3_-ZrO_2_ (FGM), indicating the good biocompatibility of FGM [31]. A glass (Na_2_O-SiO_2_-B_2_O_3_-CaO)-Hap-ZrO_2_ implant material showed better bonding with bone than a titanium implant material after a 3-month implantation period in the leg bone of a canine [32]. Li et al. reported that the glass-zirconia material exhibited good bioactivity and no cytotoxicity [33]. Very recent studies reported a densified graded glass-zirconia composition with promising mechanical properties and esthetics [10, 11, 34]. However, the biocompatibility of the graded glass-zirconia composition was not reported.

## Conclusions

According to the oral mucous membrane irritation test in conjunction with analyses of cell viability, cell adhesion, cell morphology, and oxidative stress responses, the biocompatibility of G/Z is comparable to that of Y-TZP both before and after aging. As a dental prosthesis material, G/Z shows a promising future in clinical applications. However, this study is a preliminary report, and further in vivo and in vitro studies with more comprehensive test methods are needed to confirm the present results.

## Abbreviations

DAPI: 4′,6-Diamidino-2-phenylindole dihydrochloride
F-actin: Filamentous actin
FGM: HAp-Al_2_O_3_-ZrO_2_
G/Z: Graded nano-glass/zirconia
SEM: Scanning electron microscopy
t-m: Tetragonal to monoclinic
Y-TZP: Yttrium-stabilized tetragonal zirconia polycrystal
